# Anxiety and Stress among Day Traders in Saudi Arabia

**DOI:** 10.3390/ijerph191811252

**Published:** 2022-09-07

**Authors:** Khalid A. Bin Abdulrahman, Abdulaziz Yahya Alsharif, Abdulrahman Bandar Alotaibi, Abdulrahman Ali Alajaji, Abdullah Ali Alhubaysh, Abdulrahman Ibrahim Alsubaihi, Nahaa Eid Alsubaie

**Affiliations:** 1 Department of Medical Education, College of Medicine, Imam Mohammad Ibn Saud Islamic University, Riyadh 13317, Saudi Arabia; 2 Department of Medicine, College of Medicine, Imam Mohammad Ibn Saud Islamic University, Riyadh 13317, Saudi Arabia; 3 Department of Mathematics, Alkhurmah University College, Taif University, Taif 21974, Saudi Arabia

**Keywords:** day traders, stress, work stress, anxiety, mental health, Saudi Arabia

## Abstract

Background: People nowadays are more concerned about their financial status and how to improve their quality of life; one method is day trading. This study aims to investigate the association between stress or anxiety and day trading among day traders in Saudi Arabia. Methods: We collected the data through DASS-21, a set of three self-report scales designed to measure the emotional states of depression, anxiety, and stress. It tells if the person has mild, moderate, severe, or extremely severe emotional status. Our study will focus on two domains: stress and anxiety. Day traders scoring between 0 and 7 on the anxiety scale were classified as normal anxiety. Scoring between 8 and 9 on the anxiety scale, mild anxiety, and between 10 and 14 on the anxiety scale as moderate anxiety. Those scoring between 15 and 19 were classified as severe, and those scoring >20 as extremely severe. Results: Our results showed that out of 387 valid surveys, day traders scoring < 14 on the stress scale were classified as everyday stress (N = 249, 64.3%), and those scoring between 15 and 18 as mild (N = 49, 12.7%) and those scoring between 19 and 25 as moderate (N = 46, 11.9%), those scoring between 26 and 33 as severe (N = 34, 8.8%), and those scoring > 34 were classified as extremely severe (N = 9, 2.3%). Conclusions: The prevalence of anxiety and stress is considerable among day-traders. Therefore, it is fundamental to develop more effective health promotion strategies for the target population to make them aware of and learn how to control and prevent these harmful emotional feelings.

## 1. Introduction

Little is known about the occupational health of Saudi Arabian workers, especially day traders. The world faces a growing financial stress problem, leading to psychological disorders such as anxiety and depression [1].

Day trading is defined as buying and selling a stock or any financial derivatives to make a profit in a short period. Rapid fluctuations in the share prices make the field highly competitive and stressful, which puts day traders under pressure [2]. Day trading may negatively affect day traders’ health and emotional responses by making them more anxious and s tressed. Anxiety interferes with adapting to everyday tasks, such as employment, social relationships, and trading [3].

The body responds to physical, mental, or emotional stimuli. Stress manifests as a feeling of helplessness, uncertainty of oneself, lack of sufficient strength in the face of external factors, and exaggeration of their potency and power [4]. What initiates stress in humans is variable due to social and economic status and genetic makeup; however, the lack of control and uncertainty is always distressing. This is what day traders experience since they have no control over the financial market.

More than three-quarters of adults report physical or emotional stress symptoms, such as headaches, fatigue, or changes in sleeping habits. Therefore, it is no secret that trading is stressful. In fact, according to *Business Insider*, it is the second most stressful job on Wall Street after investment banking [5].

Anxiety disorders are the most common psychiatric disorders [6]. Anxiety is an emotional state that arises in situations of imminent danger and is manifested in the expectation of unfavorable events. Most people feel anxious or scared in their daily lives. The value of this research is the documentation of the prevalence of stress and anxiety among day traders in Saudi Arabia. There is not enough literature discussing the mental health of this population, especially during the last decade, as many new day traders have joined the market, according to the Saudi capital market authority [7]. We aim to analyze whether stress and anxiety are widespread in Saudi day traders. If this is confirmed, the research community should investigate further and find solutions. We highlighted the group day traders because, in 2006, one of the unforgettable Saudi stock market crashes [8] happened, leaving behind frustration and misery. However, no clinical study has documented this history; hence, we did this research as a beginning. We assumed that day traders are more prone to anxiety and stress on a daily bases. The current study examines the status of anxiety and stress among day traders in Saudi Arabia.

## 2. Materials and Methods

### 2.1. Study Designs

This is a cross-sectional study conducted from November to December 2020. The study was conducted according to the guidelines of the Declaration of Helsinki and approved by the Institutional Review Board of the Imam Mohammad Ibn Saud Islamic University IRB committee; project number 112-2020, dated 30 November 2020.

### 2.2. Participants & Sampling

The sampling study targeted Saudi and non-Saudi, male and female, day traders (whose primary jobs were day trading) aged 18 years and older living in Saudi Arabia. Day traders who are not from Saudi Arabia and long-term investors were excluded. The participant’s contact information was obtained through the official Saudi Telecom database, which clearly explained that communication with them was purely for scientific purposes and that all data on their identities would remain confidential. A cluster sample covering all regions in Saudi Arabia, followed by a simple random sampling of those randomly selected participants from different parts of Saudi Arabia, was applied using a computer-generated system to ensure that it represents the whole country. A total of 387 participants completed the online questionnaire out of 470 invited participants. That makes the response rate 82.3%.

### 2.3. Study Questionnaire

The DASS-21 questionnaire measures depression, anxiety, and stress [9]. The questionnaire consists of three divisions. The first division comprises socio-demographic data, including age, sex, level of education, residence, buying and selling of shares on the same day (day trading), and chronic diseases.

The second division covers the stress scale questions of the DASS-21, which contains seven items. The third division is devoted to studying the applicability of anxiety to participants; seven items were included from DASS-21.

The rating scale is as follows for both stress and anxiety:

0 Does not apply to me at all;

1 Applies to me to some degree or some of the time;

2 Applies to me to a considerable degree or most of the time;

3 Applies to me very much or most of the time.

Scores on the DASS-21 are multiplied by 2 to calculate the final results, and scoring instructions for severity labels (normal, moderate, severe) are as follows.

For stress: 0–14 is considered normal, 15–18 is mild, 19–25 is moderate, 26–33 is severe, and more than 34 is extremely severe.

For anxiety: 0–7 is considered normal, 8–9 is mild anxiety, 10–14 is moderate, 15–19 severe, and a score of more than 20 is extremely severe anxiety. The participants consented that all information would be used for research purposes and were informed about the purpose of the study. The confidentiality of the data was also ensured.

### 2.4. Statistical Data Analysis

The factorability of the data was checked using Bartlett’s sphericity test, which is highly significant at a 5% level with a *p*-value < 0.001. Exploratory factor analysis was used for dimension reduction. We used the comparison data method and the scree plot method to determine the number of factors. Both methods resulted in similar results. Two domains re-identified dimension reduction by factor analysis. These domains can be classified as “stress” and “anxiety”. The stress domain consists of seven items, and the anxiety domain consists of seven items. Composite reliability (CR) and Cronbach’s alpha were applied to measure reliability, construct validity, and confirm data reliability before making any advanced analysis.

Both domains showed good internal consistency, although the reliability of the “stress” domain (Cronbach’s α = 0.84) was slightly higher than that of the “anxiety” domain (Cronbach’s α = 0.82), and the CR was over 0.70, which is considered acceptable composite reliability. See Table 1.

Finally, a Varimax rotation produced the final rotation factor solution. Items were classified as loading on a factor if they had loadings equal to or greater than 0.3 on a factor. Furthermore, the average variance extracted (AVE) was used to identify variation in the latent variables by random measurement error. Therefore, the AVE estimates of the average should range from 0.5 or higher. The AVE results in this study were achieved from 0.5 to 0.77, which exceeds the lowest recommended requirements, as shown in Table 1. One of the other recommended criteria measurements for discriminant validity is the maximum shared variation (MSV), which must achieve less than the AVE result [10]. The MSV results showed that the measurement model is valid.

The convergent and discriminant validity and reliability results revealed that the convergent validity met the requirement level, as shown in the Table above (Table 1). Furthermore, there was adequate evidence that CR and Cronbach’s alpha exceeded the minimum required level of 0.60, which improved the model’s reliability. We checked the common-method bias (CMB) using Harman’s single factor test for stress and anxiety factors. The results showed that the total variance explained by the stress factor is 51.23, slightly exceeding 50%, and less than 50% for the anxiety factor, where the total variance explained is 49.44%. The stress scale was validated using the DASS-21 Stress Scale. The interpretation of the scores on the DASS-21 scale was as follows: scores of 0–14 Normal, 15–18 Mild, 19–25 Moderate, 26–33 Severe, and >34 Extremely Severe. The anxiety scale was validated using the DASS-21 Anxiety Scale. The interpretation of the scores on the DASS-21 scale was as follows: mean scores of 0–7 = normal, 8–9 Mild, 10–14 Moderate, 15–19 Severe, and >20 extreme severe. IBM SPSS V27 (IBM, Armonk, NY, USA) was used for data analysis, and the statistical significance alpha was considered at the 0.050 level. Excel was used to create figures and depictions. The correlation coefficient was computed using the Pearson correlation test to investigate the correlation. One-way ANOVA was used to compare stress levels between different levels of education. Levene’s test was used to check the assumption of homogeneity of variances. The independent t-test was used to compare the stress score between those with/without chronic diseases. The normality of anxiety and stress scores were tested using skewness and kurtosis.

## 3. Results

The characteristics of the study population are shown in Table 2. In total, 93% of the day traders were male, the majority were located in the central region of Saudi Arabia (85%), and 55.6% belonged to the 18–35-year age group. Most day traders have completed university-level education (64.3%). More than 17% of the day traders were found to have chronic diseases.

The stress indicators of the Saudi day traders are shown in Table 3. Moreover, the stress and anxiety indicators of the Saudi traders are presented in Table 4.

The item-total correlations for each item ranged between r = 0.21 and r = 0.60. The correlation matrix of the 14 items of the Stress and Anxiety Scale is shown in (Table 5).

**Figure 1:**
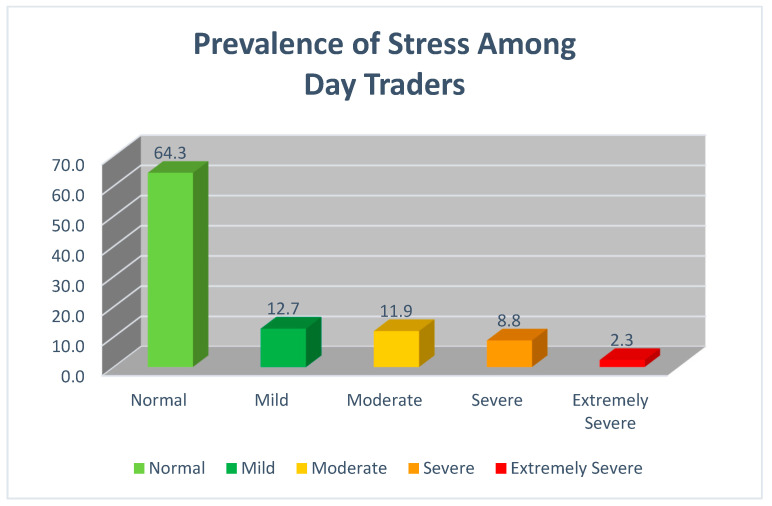
Prevalence of Stress Among Day Traders.

Day traders scoring < 14 on the Stress Scale (displayed in Figure 1) were classified as everyday stress (N = 249, 64.3%), those as mild (N = 49, 12.7%), and those as moderate (N = 46, 11.9%), those as severe (N = 34, 8.8%), and those as extremely severe (N = 9, 2.3%).

**Figure 2:**
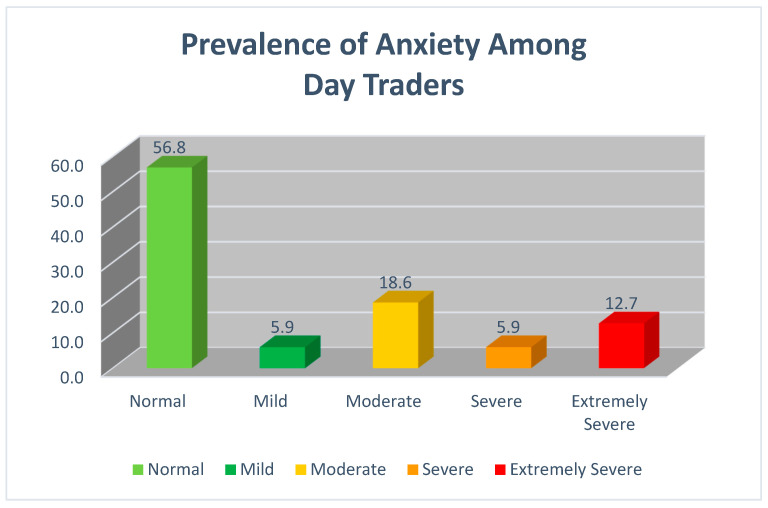
Prevalence of Anxiety Among Day Traders.

Day traders were classified (displayed in Figure 2) as normal anxiety (N = 220, 56.8%), as mild anxiety (N = 23, 5.9%), as moderate anxiety (N = 72, 18.6%), as severe (N = 23, 5.9%), and as extremely severe (N = 49, 12.7%). See Figure 2.

The normality of anxiety and stress scores were between ±3, and the variables were normally distributed (Table 6).

Various levels of education were associated with stress scores, as shown in the one-way ANOVA (*F*(3, 383), *p* = 0.033). The public university group (M = 11.32, SD = 8.04) reported a significantly lower stress score level (*p* = 0.025) than the other group (M = 18.35, SD = 7.98), with a mean difference of 7.04 as revealed by post hoc pairwise comparisons using Tukey test.

According to Levene’s test, the assumption of homogeneity of variances was met, F = 0.20, *p* = 0.654. The 66 participants in the chronic disease group have significantly higher stress scores (M = 16.39, SD = 9.52) than the 321 participants in the group without chronic disease (M = 11.96, SD = 8.89). The result showed a significant difference, t(385) = −3.65, *p* < 0.001.

Similarly, there was a significant difference in the mean anxiety score between the participants in the chronic disease group (M = 10.88, SD = 9.20) compared to the 321 participants in the no chronic disease group (M = 8.16, SD = 8.44). The Levene test showed that the variances are assumed to be equal, F = 1.87, *p* = 0.172. The results of the t-test were significant, t(385) = −2.35, *p* = 0.019.

A Chi-square test was conducted to check the associations between stress determinants, demographics, and medical conditions, and the significant associations were highlighted in Bold (Table 7).

The Chi-square test was also conducted to check the associations between stress and anxiety levels and demographics, and medical conditions and the significant associations were highlighted in Bold (Table 8).

## 4. Discussion

The results of this cross-sectional study in Saudi Arabia that investigates the prevalence of stress and anxiety among day traders indicate that day traders are not immune to psychological instability (stress and anxiety). Our study identified stress and anxiety as two domains within day trading. Of 387 day-traders, 34 (8.8%) have severe stress, and 49 (12.7%) report extremely severe anxiety. In our findings, 9 (2.3%) day traders reported extreme stress, which is not close to the percentage of day traders who reported “very high” or “extremely high” stress levels in a study on work stress and performance among financial traders [11,12].

“Some traders have snapped; this business is not for everyone,” a trader admitted. In contrast, another day trader said, “You make money based on risk, and there is stress associated with this risk.”. These statements point to the general high-stress levels [12]. Furthermore, the relationship between financial aspects of life and psychological consequences such as depression, stress, and post-traumatic stress disorder (PTSD) have been extensively documented [3,12,13], inconsistent with our results.

Because trading is likely to involve higher brain functions, such as long-term planning, numerical computation, and logical reasoning, our findings are consistent with current neuroscientific evidence that emotional responses such as fear and greed (e.g., responses mediated by the amygdala) can make traders anxious and stressed during the trading process [13]. Some researchers studied the similarities and differences between traditional gambling and day trading in South Australia. They found that most day traders were also engaged in conventional forms of gambling [14]. Other literature shows that gambling and stress are correlated [15]. This does not necessitate that day traders be gamblers, but they sometimes behave like gamblers, leading to psychological stress and addiction.

There is a type of trading referred to as “margin trading”. Margin allows traders to amplify their purchasing power to leverage into more prominent positions than their cash positions would otherwise allow. When borrowing money from the broker to trade in larger quantities, traders can amplify returns and potential losses [16]. This type of trading has a kind of gambling behavior in the way that people who practice this type of trading act as gamblers by increasing the risks, in turn, to achieve very high possible profits even if they do not have the cash to do so, and if they lose, they have an overwhelming debt that can lead to depression, anxiety, and even hospitalization. Literature documented the relationship between margin trading in the stock markets and stroke hospitalizations in Taiwan; they found that the increase in margin trading in the Taiwan stock markets was associated with more significant stroke hospitalizations, specifically the third and sixth days after the increase in margin trading [17].

In our findings, men outnumbered women by nine times, but there is no significant trend for men to experience more stress than females [12]. On the other hand, women tend to trade less than men, and older people tend to trade less than younger people, according to a study published by Andrew [3], which is consistent with our findings in which 55.6% of day traders were aged between 18–35.

Almost two-thirds of day traders have completed university-level education; this makes them aware and knowledgeable about assessing risk and benefits more adequately for being engaged in this field. Investors with academic degrees trade stocks more actively, which puts them under pressure to make decisions and think faster [18]. However, our results showed that anxiety level is significantly associated with education.

Some day traders were found to have diabetes. Evidence shows that anxiety and diabetes are significantly correlated in that diabetes is associated with an increased chance of having anxiety disorders and elevated anxiety symptoms. Evidence supports the idea that anxiety may increase the risk of developing diabetes and not the opposite. Post-traumatic stress disorder, for example, is believed to be a risk factor for developing type 2 diabetes. Another piece of evidence suggests that psychosocial stress may influence the onset of type 1 diabetes [19]. However, our findings are not significant for the association between diabetes and stress or anxiety; this could be due to the low number of participants who have diabetes.

Furthermore, there were day traders who had hypertension. The literature suggests that there is an association between stress and hypertension. Adults with hypertension were more likely to develop stress, which can be implemented the other way around, where patients with recurrent stress have a higher risk of developing hypertension; this deduction was indicated in patients who did not have any risk factors related to hypertension. From a physiological point of view, hypertension and stress can be explained in a way that stress, defined as a negative emotion, has psychological (e.g., tension, worry) and physical (e.g., palpitations, dry mouth) characteristics. It leads to overactivity of the mind and emotions, ultimately having physiological effects on the heart through autonomic stimulation, thus causing increased blood pressure [20].

In addition, stress is commonly experienced through anxiety, mediated by the hypothalamic–pituitary–adrenal (HPA) axis that controls and increases the production of circulating catecholamine levels, which activates the sympathetic nervous system and increases the blood pressure of the sympathetic nervous system. Changes in circulating catecholamines, autonomic mechanisms that lead to insulin resistance, hypertension, inflammation, and endothelial dysfunction, can be attributed to cardiovascular diseases [21].

## 5. Limitations

This survey highlights ongoing methodological limitations, including the cross-sectional study design that makes causation inference difficult. Another issue for all studies that use a self-reported questionnaire is confounding factors, especially anxiety, stress, or emotions, because they can be affected by multiple factors. Furthermore, it was challenging to reach the participants or the literature related to our research, especially since we did not find published papers addressing mental health and day trading in Saudi Arabia; furthermore, market performance might have influenced the participant’s responses to the survey.

## 6. Conclusions

Ultimately, this study denoted the issue of stress and anxiety among day traders in Saudi Arabia. Future research on traders’ stress should investigate the behavior of day traders, the role of personality, and the impact of education, gender disparities, and age in day trading. Screening for mental well-being should also target day traders, who are at high risk of stress and anxiety that can lead to depression and other mental disorders.

## Figures and Tables

**Table 1:** Reliability and Construct Validity.

Estimate	Construct	Estimate λ	Cronbach’s α	AVE *	CR **	MSV ***	CV ****	DV *****
Stress	S1	0.715	0.84	0.51	0.88	0.46	Yes	Yes
	S2	0.675						
	S3	0.778						
	S4	0.813						
	S5	0.725						
	S6	0.707						
	S7	0.572						
Anxiety	A1	0.741	0.82	0.50	0.87	0.46	Yes	Yes
	A2	0.764						
	A3	0.748						
	A4	0.728						
	A5	0.729						
	A6	0.726						
	A7	0.426						

* Average variance extracted (AVE), ** Composite reliability (CR), *** Maximum shared variance (MSV), **** Convergent Validity, ***** Discriminant Validity.

**Table 2:** Characteristics of the Saudi day trader sample (N = 387).

			Number	%
**Gender**		Female	27	7.0%
		Male	360	93%
**Age**		18–35	215	55.6%
		36–55	137	35.4%
		≥56	35	9.0%
**Level of education**		Below university	73	18.9%
		University	249	64.3%
		Post-Graduate education	48	12.4%
		Others	17	4.4%
**Residence location**		Southern region	28	7.2%
		Northern region	21	5.4%
		Eastern Region	85	52.2%
		Western Region	51	13.2%
		Central Region	202	85.0%
**Chronic diseases:**				
	**Heart disease**	No Yes	381 6	98.4% 1.6%
	**Diabetes**	No Yes	354 33	91.5% 8.5%
	**Hypertension**	No Yes	362 25	93.5% 6.5%
	**Others (Panic, Asthma, Migraine, etc.)**	No Yes	371 16	95.9% 4.1%

**Table 3:** Stress indicators of the Saudi day traders.

	1. Not Applicable	2. Somewhat	3. Most of the Time	4. All the Time
I find it difficult to calm down after anger	144 (37.2%)	141 (36.2%)	54 (14.0%)	48 (12.4%)
I tend to overreact to circumstances and events	128 (33.1%)	174 (45%)	65 (16.8%)	20 (5.2%)
I felt like I was consuming too much nervous energy and consuming too much of my nervous tension	118 (30.5%)	160 (41.3%)	72 (18.6%)	37 (9.6%)
I felt disturbed and upset	124 (32.0%)	164 (42.4%)	78 (20.2%)	21 (5.4%)
I find it difficult to relax and rest	171 (44.2%)	139 (35.9%)	52 (13.4%)	25 (6.5%)
I couldn’t bear anything turning me back from what I wanted to do	145 (37.5%)	142 (36.7%)	56 (14.5%)	44 (11.4%)
I felt inclined to get angry quickly	257 (66.4%)	88 (22.7%)	12 (3.1%)	30 (7.8%)

**Table 4:** Stress and anxiety indicators of the Saudi day traders.

	1. Not Applicable	2. Somewhat	3. Most of the Time	4. All the Time
I felt dryness in my throat	275 (71.1%)	74 (19.1%)	15 (3.9%)	23 (5.It%)
I felt hard to breathe	261 (67.4%)	91 (23.5%)	10 (2.6%)	25 (6.5%)
I felt trembling in the hands	236 (61.0%)	97 (25.1%)	19 (4.9%)	35 (9.0%)
I was afraid of situations where I might lose control of my nerves and cause myself the embarrassment.	265 (68.5%)	83 (21.4%)	18 (4.7%)	21 (5.4%)
I felt on the verge of falling into sudden terror for no reason	237 (61.2%)	96 (24.8%)	18 (4.7%)	36 (9.3%)
I felt my heart beat faster without any physical effort	227 (58.7%)	103 (26.6%)	17 (4.4%)	40 (10.3%)

**Table 5:** Correlation matrix of the 14 items of the Stress and Anxiety Scale.

	1	2	3	4	5	6	7	8	9	10	11	12	13	14
1	1	0.318 **	0.463 **	0.584 **	0.469 **	0.391 **	0.318 **	0.213 **	0.264 **	0.397 **	0.294 **	0.329 **	0.456 **	0.354 **
2		1	0.522 **	0.437 **	0.354 **	0.465 **	0.301 **	0.251 **	0.297 **	0.330 **	0.301 **	0.233 **	0.244 **	0.418 **
3			1	0.601 **	0.415 **	0.436 **	0.407 **	0.244 **	0.323 **	0.390 **	0.271 **	0.264 **	0.337 **	0.403 **
4				1	0.497 **	0.515 **	0.345 **	0.338 **	0.344 **	0.416 **	0.364 **	0.324 **	0.338 **	0.357 **
5					1	0.485 **	0.406 **	0.296 **	0.320 **	0.437 **	0.425 **	0.355 **	0.502 **	0.375 **
6						1	0.221 **	0.319 **	0.301 **	0.441 **	0.380 **	0.369 **	0.322 **	0.342 **
7							1	0.351 **	0.289 **	0.366 **	0.273 **	0.239 **	0.331 **	0.237 **
8								1	0.565 **	0.427 **	0.462 **	0.478 **	0.409 **	0.214 **
9									1	0.531 **	0.444 **	0.458 **	0.421 **	0.238 **
10										1	0.443 **	0.484 **	0.451 **	0.271 **
11											1	0.430 **	0.528 **	0.190 **
12												1	0.440 **	0.224 **
13													1	0.281 **
14														1

Note: **, *p* < 0.01.

**Table 6:** The normality of anxiety and stress scores.

	Min	Max	M	SD	Skewness	Kurtosis
Stress score	0.00	40.00	12.72	9.14	0.71	0.04
Anxiety score	0.00	40.00	8.62	8.63	1.32	1.27

Pearson correlation test showed a significant positive correlation between anxiety and stress scores, r(385) = 0.675, *p* < 0.001.

**Table 7:** Stress determinants to sex and medical conditions.

	Age	Education	Gender	Heart Disease	Diabetes	Hypertension	Other
	χ^2^ (*p*)	χ^2^ (*p*)	χ^2^ (*p*)	χ^2^ (*p*)	χ^2^ (*p*)	χ^2^ (*p*)	χ^2^ (*p*)
I find it difficult to calm down after anger	9.43 (0.151)	**39.40 (<0.001)**	1.96 (0.580)	0.16 (0.983)	4.71 (0.194)	**19.68 (<0.001)**	1.19 (0.756)
I tend to overreact to circumstances and events	10.79 (0.095)	**20.94 (0.013)**	3.05 (0.383)	4.21 (0.239)	0.84 (0.839)	**10.12 (0.018)**	7.73 (0.052)
I felt like I was consuming too much nervous energy	**16.92 (0.010)**	**21.01 (0.013)**	3.87 (0.276)	3.19 (0.371)	5.53 (0.137)	5.34 (0.148)	**10.04 (0.018)**
I felt disturbed and upset	6.73 (0.347)	8.34 (0.500)	0.33 (0.954)	5.72 (0.126)	3.19 (0.363)	**10.43 (0.015)**	**10.43 (0.015)**
I find it difficult to relax and rest	8.51 (0.203)	**27.56 (0.001)**	1.42 (0.700)	7.81 (0.050)	1.80 (0.614)	**9.34 (0.025)**	1.57 (0.667)
I couldn’t bear anything turning me back from what I wanted to do	**25.98 (<0.001)**	4.79 (0.852)	1.81 (0.614)	3.23 (0.358)	5.12 (0.163)	**11.56 (0.009)**	7.33 (0.062)
I felt inclined to get angry quickly	**24.58 (<0.001)**	**33.68 (<0.001)**	2.15 (0.541)	2.90 (0.407)	3.00 (0.391)	**21.57 (<0.001)**	7.06 (0.070)

The chi-square results in Table 7 show that stress levels have an association with education (*p* = 0.007), hypertension (*p* = 005), and other medical conditions (*p* = 006); in addition to that, anxiety levels have a significant association with education (*p* = 043).

**Table 8:** Stress and Anxiety Levels in Relation to Gender, Age, Education, and Medical Conditions.

	Age	Education	Gender	Heart Disease	Diabetes	Hypertension	Other
	χ^2^ (*p*)	χ^2^ (*p*)	χ^2^ (*p*)	χ^2^ (*p*)	χ^2^ (*p*)	χ^2^ (*p*)	χ^2^ (*p*)
Stress Level	16.34 (0.38)	**27.27 (0.007)**	1.47 (0.831)	1.38 (0.848)	4.22 (0.377)	**15.05 (0.005)**	**14.30 (0.006)**
Anxiety Level	10.04 (0.262)	**21.58 (0.043)**	3.14 (0.535)	2.92 (0.571)	1.17 (0.882)	7.06 (0.133)	9.05 (0.060)

## Data Availability

All data supporting the study findings are contained in this manuscript.
